# Photocatalytic Reduction of CO_2_ with N-Doped TiO_2_-Based Photocatalysts Obtained in One-Pot Supercritical Synthesis

**DOI:** 10.3390/nano12111793

**Published:** 2022-05-24

**Authors:** Óscar R. Andrade, Verónica Rodríguez, Rafael Camarillo, Fabiola Martínez, Carlos Jiménez, Jesusa Rincón

**Affiliations:** Department of Chemical Engineering, Faculty of Environmental Sciences and Biochemistry, University of Castilla-La Mancha, Av. Carlos III, s/n, 45071 Toledo, Spain; oscarramiro.andrade@uclm.es (Ó.R.A.); veronica.rodriguez@uclm.es (V.R.); fabiola.martinez@uclm.es (F.M.); carlos.jimenez@uclm.es (C.J.); jesusa.rincon@uclm.es (J.R.)

**Keywords:** titania, nitrogen doping, carbon nanotubes, reduced graphene oxide, carbon dioxide conversion

## Abstract

The objective of this work was to analyze the effect of carbon support on the activity and selectivity of N-doped TiO_2_ nanoparticles. Thus, N-doped TiO_2_ and two types of composites, N-doped TiO_2_/CNT and N-doped TiO_2_/rGO, were prepared by a new environmentally friendly one-pot method. CNT and rGO were used as supports, triethylamine and urea as N doping agents, and titanium (IV) tetraisopropoxide and ethanol as Ti precursor and hydrolysis agent, respectively. The as-prepared photocatalysts exhibited enhanced photocatalytic performance compared to TiO_2_ P25 commercial catalyst during the photoreduction of CO_2_ with water vapor. It was imputed to the synergistic effect of N doping (reduction of semiconductor band gap energy) and carbon support (enlarging e^−^-h^+^ recombination time). The activity and selectivity of catalysts varied depending on the investigated material. Thus, whereas N-doped TiO_2_ nanoparticles led to a gaseous mixture, where CH_4_ formed the majority compared to CO, N-doped TiO_2_/CNT and N-doped TiO_2_/rGO composites almost exclusively generated CO. Regarding the activity of the catalysts, the highest production rates of CO (8 µmol/gTiO_2_/h) and CH_4_ (4 µmol/gTiO_2_/h) were achieved with composite N^1^/TiO_2_/rGO and N^1^/TiO_2_ nanoparticles, respectively, where superscript represents the ratio mg N/g TiO_2_. These rates are four times and almost forty times higher than the CO and CH_4_ production rates observed with commercial TiO_2_ P25.

## 1. Introduction

The use of photocatalytic technology to chemically reduce carbon dioxide (CO_2_) into hydrocarbons not only transforms this greenhouse gas into reusable fuel, but also helps alleviate global warming [1]. However, CO_2_ is an extremely stable compound, and photocatalytic CO_2_ reduction with solar light still remains a challenge, mainly because of low solar energy conversion efficiency, backward reaction phenomenon, uncontrolled product selectivity and rapid electron-hole recombination rate of the photocatalyst [2].

As in many other environmental and energy applications, titanium dioxide has been the photocatalyst more widely used for the conversion of CO_2_ to fuel, mainly due to its photoactivity, high stability, low cost, and safety [3,4]. However, its application is limited because of its relative wide band gap (3–3.2 eV) and rapid recombination rate of photo-induced electron-hole pairs [5]. To overcome these drawbacks, different strategies have been proposed, such as doping with transition metal cations [6], using enhanced geometries [7] or photocatalyst supporting on carbon materials [8,9].

Non-metal doping is another approach suggested to improve TiO_2_ performance. Compared with metal doping, non-metal dopants lead to catalysts with higher photo-stability, less environmental contamination, and lower cost [10]. Particularly, doping with N atoms into the TiO_2_ lattice structure leads to the generation of N 2p energy levels near to the valence band (VB) of TiO_2_, thus reducing its band gap and extending semiconductor light absorption to the visible spectrum [5]. Moreover, N atoms also prevent the recombination of charge carriers in N-doped TiO_2_ and can form metastable centers due to their stability, low ionization potential and having an atomic radius comparable with oxygen [5]. However, the increases achieved in visible light absorption and lifetime of the electron-hole pairs photogenerated with this catalytic material are still limited.

Fortunately, this limitation can be alleviated by supporting the upgraded semiconductor on carbon nanotubes (CNTs) or reduced graphene oxide (rGO), since both carbon materials can increase the generation of excitons and reduce their recombination rates; thereby promoting a cooperative and synergistic effect that can enhance the overall efficiency of the photocatalytic process [11,12]. Further, CNT and rGO can provide large specific surface areas for photocatalysis [13]. Even more, the conductive structure of CNT and rGO scaffolds is believed to favor the separation of the photo-generated electron-hole pairs through the formation of heterojunctions (Scottky barrier) at the TiO_2_/CNT or TiO_2_/rGO interfaces [13,14]. Finally, from a morphologic point of view, the support of TiO_2_ over CNT and rGO avoids particle agglomeration [15] and, thus, improves the homogeneous dispersion of this semiconductor and the availability of most active centers.

Considering the above, the combination of N-doped TiO_2_ with carbon supports has been recently proposed and reported in literature [16,17]. However, for these complex systems, the joint effect of N-doping and carbon supporting of TiO_2_ on enhancing of sunlight absorption and electron-hole separation still remains unclear [10]. Particularly, there is controversy regarding which type of carbon support can be more beneficial for the photocatalytic reduction of CO_2_ [18,19]. Thus, the main objective of this work has been to develop N-doped TiO_2_ nanoparticles supported on CNT and rGO and to investigate the influence of the carbon support of the composites on both physicochemical properties and catalytic performance in CO_2_ photoreduction. To our best knowledge, this type of analysis is here tackled for the first time.

Regarding the synthesis of N-doped TiO_2_ nanostructures, it can be accomplished by different methods, such as sputtering, sol-gel process, anodic oxidation, microwave, hydrothermal, microemulsion, chemical vapor deposition, solvothermal, and electrospinning, among other processes. However, these processes usually suffer from scale-up problems and are not environmentally friendly [20]. Providentially, both obstacles may eventually vanish if the catalyst synthesis is carried out in a supercritical CO_2_ (scCO_2_) medium, i.e., in CO_2_ at pressure and temperature conditions above critical point [20]. Moreover, there are several favorable physical properties of the supercritical fluids, such as low viscosity, “zero” surface tension and high diffusivity [21], that enable them to produce superior, ultrafine, and uniform nanomaterials at appropriate operating conditions [22,23]. In addition, scCO_2_ can be removed completely from the products by venting. Thus, no drying process is required, and the porous structure can be maintained without collapsing of the nanostructure. Further, scCO_2_ can be easily recycled after decompressing and potential scale-up is feasible.

In view of the above, in this study we report for the first time on the one-pot synthesis of a series of N-doped TiO_2_ nanoparticles and N-doped TiO_2_-based composites (N/TiO_2_/CNT and N/TiO_2_/rGO) in scCO_2_ medium. For the nanomaterials synthesis process, titanium (IV) tetraisopropoxide (TTIP) and triethylamine (TEA) or urea were used as precursors of TiO_2_ and N, respectively, and ethanol was used as the hydrolytic agent, which decomposes to water molecules that react with TTIP to produce TiO_2_. CNT or rGO were also used in the synthesis of the composites. The characteristics of the synthesized materials, and their activities in CO_2_ photoreduction with water vapor under simulated solar light irradiation, were investigated. On this base, efficient photocatalytic conversion of CO_2_ was demonstrated. Further, the differences in selectivity and production rates of CO and CH_4_ obtained with each catalyst are explained in terms of the physicochemical properties of the catalytic material analyzed.

## 2. Materials and Methods

### 2.1. Chemicals

Multi-walled carbon nanotubes (MWCNT, purity > 98%) were supplied by Sigma- Aldrich (Darmstadt, Germany). For graphene oxide synthesis, potassium permanganate (KMnO_4_, analytical reagent), hydrochloric acid (HCl, 37%) and sulfuric acid (H_2_SO_4_, 30 wt. %) were provided by Panreac (Madrid, Spain), graphite powders by Merck (Darmstadt, Germany), and hydrogen peroxide (H_2_O_2_, 30 wt. %) by Scharlau (Barcelona, Spain). For the preparation of nitrogen-doped catalysts (N-doped TiO_2_, N-doped TiO_2_/CNT and N-doped TiO_2_/rGO), titanium (IV) tetraisopropoxide (TTIP, 98+%) was provided by Across Organics (Madrid, Spain), ethanol (analytical reagent) and triethylamine (TEA, EssentQ) by Scharlau (Barcelona, Spain), and urea (99–100.5%) by Sigma-Aldrich (Darmstadt, Germany).

CO_2_ (purities > 99.9% for synthesis and 99.998% for photocatalytic studies) was used as received from Nippon (Madrid, Spain) and Contse (Madrid, Spain).

### 2.2. Synthesis of Catalysts

The synthesis of rGO was performed by the Hummers’ method, as described in a previous work [9].

In the case of N-doped TiO_2_ nanoparticles, the one-pot reaction was developed by adding the titanium precursor (TTIP, 1.39 g), the hydrolysis agent (ethanol, 8 mL) and 1–4 mL TEA (0.8–2.9 g) or urea (0.3 g) as nitrogen precursor [20]. The reactions took place in a stainless-steel reactor (volume 100 mL) using scCO_2_ as solvent at a pressure of 200 bar and a temperature of 300 °C. This procedure was described in more detail in previous works [24]. Later, the solid obtained was dried at 105 °C for 24 h and calcined at 400 °C for 3 h. The catalyst was named N^X^/TiO_2,_ X being the N content (mg N/g TiO_2_) in the synthetized N-doped TiO_2_ nanoparticles. All catalysts were obtained in triplicate.

A similar procedure was used for synthesizing composites of N-doped TiO_2_ over CNT or rGO (N^X^/TiO_2_/CNT and N^X^/TiO_2_/rGO, respectively). The quantities of TTIP and ethanol added were the same as those used for N-doped TiO_2_ nanoparticles but 390 mg of either CNT or GO were also aggregated, thus keeping the TiO_2_:carbon support mass ratio equal to 1 [8,9]. In this case, 1–4 mL (0.8–2.9 g) of TEA or 0.3 g of urea were employed. The conditions of the drying process of the composites were also the same. However, whereas N-doped TiO_2_/CNT composites were calcinated at 400 °C for 3 h [8], the conditions for N-doped TiO_2_/rGO composites were 500 °C for 3 h in a nitrogen atmosphere [9].

### 2.3. Characterization of Catalysts

The synthesized photocatalysts were characterized with different analytical techniques. The N content was determined in an elemental analyzer (CHNS-932, LECO, Geleen, The Netherlands). A transmission electron microscope (TEM, 2100, Jeol, Croissy-sur-Seine, France) was used to obtain information about the morphology of the catalysts. An X-ray powder diffractometer (XRD, X’Pert MDP, Phillips, Amsterdam, The Netherlands) was used to determine crystallinity, crystallite size and crystalline phases of the catalysts. The specific surface area of the powders was measured using a BET area analyzer (Nova Touch LX2, Quantachrome, Graz, Austria). The presence of certain functional groups was determined by Fourier Transform Infrared spectroscopy (FTIR) analysis with a Spectrum 100 FTIR spectroscope (Perkin-Elmer, Madrid, Spain). The X-ray photoelectron spectroscopy measurement (XPS) was made in an XPS-AES spectrometer (AXIS UltraDLD, Kratos, Manchester, UK). A diffuse reflectance UV-vis spectrophotometer (DRS, V650, Jasco, Croissy-sur-Seine, France) was employed to obtain absorbance thresholds and band gap energies. Electrochemical impedance spectroscopy (EIS) experiments were performed using a PGSTAT302N potentiostat (AUTOLAB, Utrecht, The Netherlands), a 0.1 M KHCO_3_ solution was used as electrolyte, a calomel electrode as reference electrode, a Pt electrode as counter electrode and a frequency range of 0.005–10,000 Hz.

### 2.4. Photocatalytic Reaction Tests

For all synthesized catalysts, the photocatalytic reduction of CO_2_ in gas phase with water vapor was performed, as described in previous works [8,9]. In short, the catalyst (50 mg) was immobilized in a filter and placed inside a stainless-steel reactor with a quartz window. Then, the reactor was filled with the mixture of water vapor and CO_2_ until the operating conditions were reached. Next, it was illuminated using a Xe arc lamp (450 W, Oriel, Irvine, CA, USA) with an Air Mass 1.5 Global filter to simulate sunlight. Once the experiment was finished, after 3 h of reaction, the reduction products were determined with a GC (490 Micro GC, Agilent, Santa Clara, CA, USA) connected to the reactor.

Control experiments were performed for the evaluation of photocatalysts. Results from these tests showed no appreciable amounts of reduced products in the absence of catalysts or light irradiation, illustrating that the process occurring in the reactor was photocatalytic in nature. Additionally, no reduction products were detected when introducing He into the reactor, instead of CO_2_, or when adding CO_2_ in the absence of water under light irradiation, indicating that the CO and CH_4_ originated from CO_2_ in the presence of water under light irradiation.

## 3. Results

### 3.1. N Content of the N-Doped Catalysts

In the first place, when both supports (CNT and rGO) were doped with N using 1–4 mL of TEA, a N precursor first employed in supercritical doping by Lucky and Charpentier [20], the N loads reached a maximum of 2 mg N/g CNT and 10 mg N/g rGO, respectively (Table 1). When they were doped with 0.3 g of urea (the maximum amount allowed before it precipitates from the ethanol solution in the presence of TTIP), the resulting N concentrations were 2 mg N/g CNT and 42 mg N/g rGO, respectively.

These results illustrate that larger N loads were achieved in the rGO support, regardless of the N precursor used for doping.

Regarding N-doped photocatalysts (Table 2), when they were synthesized with TEA, it can be seen that the N content in N-doped TiO_2_ nanoparticles, N-doped TiO_2_/CNT composites and N-doped TiO_2_/rGO composites was about 1 mg N/g TiO_2_. N content expressed in mg N/g TiO_2_ in composites was estimated according to the results in Table 1 for N-doped supports and considering the results in Table 2 for N-doped TiO_2_ nanoparticles.

On the other hand, it can be appreciated that the N content of the catalysts obtained with 0.3 g urea was always larger than those produced from TEA, this effect being especially noticeable in the presence of rGO.

These results demonstrated the well-known fact that the N content of N-doped TiO_2_-based catalysts largely depends on the molecular structure of the source of nitrogen and the accessibility of nitrogen atoms to react with the titania precursor and support [25]. In this sense, primary and secondary amines are likely to provide a N-richer catalyst material. For this reason, most works have used urea, since its primary amine structure is likely to introduce the highest amount of N to the catalyst. Triethylamine, as a tertiary amine, was expected to provide less nitrogen atoms to titania than urea. However, it should be noted that this does not necessarily imply that the photoactivity of TEA-doped catalysts will be lower [26,27], as will be shown below.

In the following sections the nature of the nitrogen present in the synthesized catalysts and the catalyst properties it affects will be discerned.

### 3.2. Photocatalytic Activities

**Figure 1:**
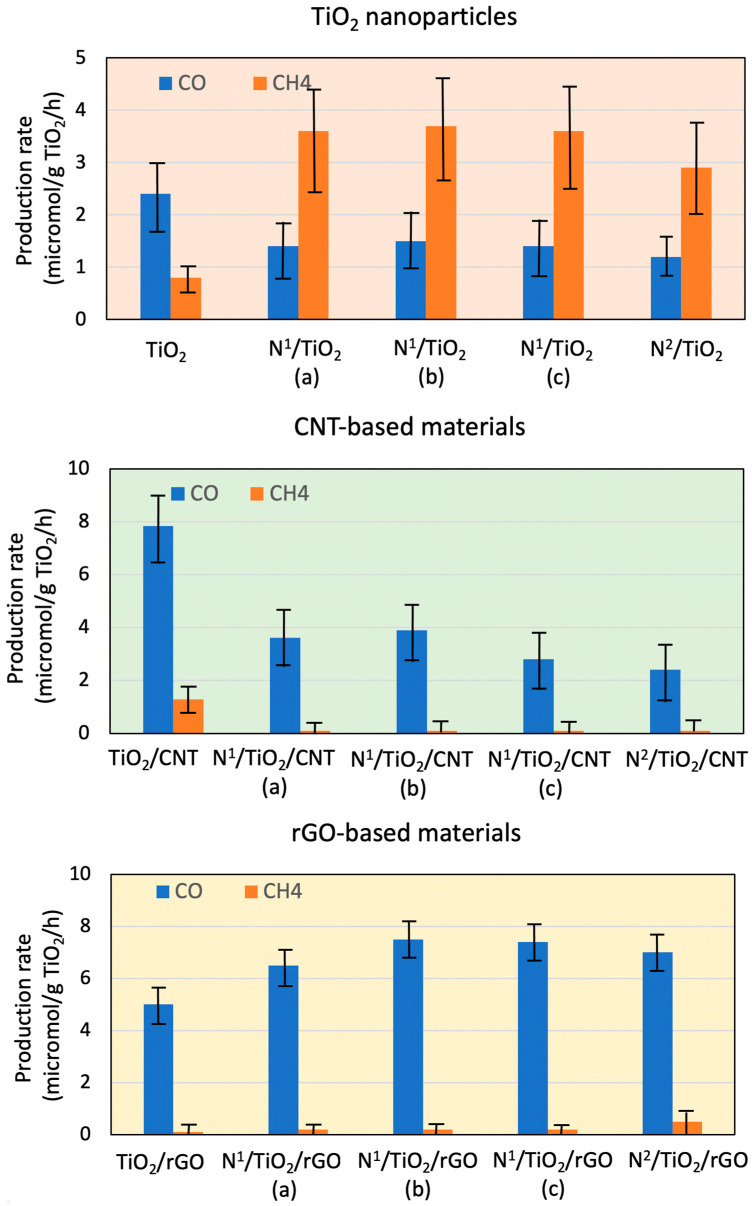
Photocatalytic production rates of different synthesized N-doped photocatalysts.

Figure 1 shows the CO_2_ conversion rates obtained with different synthesized catalysts during its photoreduction with water vapor in the presence of simulated sunlight. The average value and standard deviation of 3 replications for each material are presented. They are expressed in terms of µmol of product per hour and gram of TiO_2_, which is the photo-active species. As was the case with undoped and metal-doped TiO_2_ particles [6], TiO_2_/CNT [8], and TiO_2_/rGO composites synthesized in supercritical medium [9], the only reduction products detected were CO and methane. Moreover, all the prepared catalysts showed higher photocatalytic activity than the previous bare TiO_2_ nanoparticles synthesized in supercritical medium, indicating that the presented one-pot method for doping + synthesizing + supporting the photocatalyst is an efficient way to produce photocatalysts with higher activity than that exhibited by TiO_2_.

Previously, similar experiments with all synthesized N-doped supports were performed, with smaller CO and methane concentrations obtained than the detection limits in all cases.

Specifically, the results obtained in this work can be differentiated into 3 groups. On the one hand, the N-doped TiO_2_ nanoparticles (N^1^/TiO_2_) doubled the CO_2_ total conversion rates of non-doped TiO_2_ nanoparticles obtained at supercritical conditions (3.2 µmol products/h/g TiO_2_) (Figure 1) and tripled that of P25 (2.1 µmol products/h/g TiO_2_) [8]. Regarding the products obtained, the selectivity towards methane was greatly increased (selectivity 91%) compared to undoped TiO_2_ particles (selectivity 57%) [8]. This selectivity was calculated according to Fu et al. (2020) [28]. Next, the N-doped TiO_2_/CNT composites (e.g., N^1^/TiO_2_/CNT) exhibited lower results than those corresponding to the non-doped composites, both in total conversion (4 vs. 9.1 µmol products/h/g TiO_2_) and in selectivity towards methane (9% vs. 40%) [8]. Finally, the N-doped TiO_2_/rGO composites obtained from TEA as N precursor (N^1^/TiO_2_/rGO) showed similar selectivity towards methane as the undoped TiO_2_ catalysts supported on rGO (around 10%), but the total CO_2_ conversion rates were about 50% higher (7.7 vs. 5.1 µmol products/h/g TiO_2_) [9].

On the other hand, it can be appreciated that the results (CO_2_ conversion and selectivity towards CH_4_) were slightly lower for the catalysts with higher N content synthesized using urea as N source. Particularly, only the methane selectivity of composite N^2^/TiO_2_/rGO (synthesized from urea) (22%) was slightly higher than that of composite N^1^/TiO_2_/rGO, a composite obtained from TEA.

In conclusion, it was shown that, whereas supporting on CNT did not improve the results of TiO_2_ nanoparticles, supporting on rGO improved the CO_2_ conversion but not CH_4_ selectivity, when similar N content (in terms of mg N/g TiO_2_) was employed.

Obviously, the results obtained with CNT composites were unexpected and may be imputed to the lower crystallinity and crystal size of N-doped TiO_2_/CNT composites when compared with TiO_2_/CNT composites, as will be shown in corresponding sections. Moreover, XPS analysis suggested that N incorporation took place mainly in the bulk, but not on the surface, in N-doped TiO_2_/CNT composites, as the N-containing active sites were less accessible. Both phenomena lead to lower charge transfer and, consequently, lower photocatalytic activity.

In the following sections we will further explain these results considering the characteristics of the different catalysts. However, before this, they will be compared to those obtained in similar studies with N-doped catalysts synthesized with traditional methods. In this sense, it can be seen in Table 3 that the results from this work are far higher than those reported in the bibliography for N-doped TiO_2_ nanoparticles and N-doped TiO_2_/carbon support composites.

**Figure 2:**
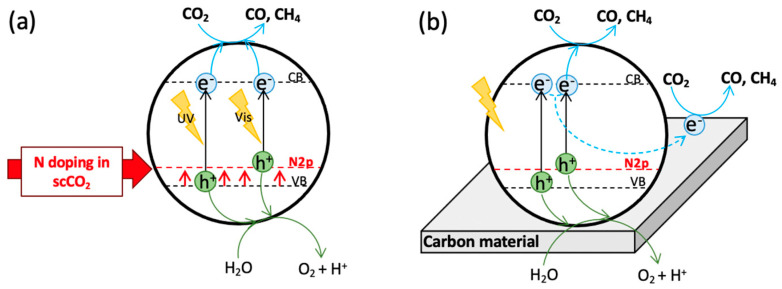
Photocatalytic mechanism: (**a**) N–doped TiO_2_ nanoparticles, (**b**) N–doped TiO_2_/carbon support composites.

The photocatalytic mechanism of N-doped TiO_2_ catalysts may be described as follows [14] (Figure 2a). The N 2p energy level, situated above the VB of TiO_2_, forms a narrower band gap than that of bare TiO_2_, which extends the absorption of N-doped TiO_2_ into the visible region. Under solar light irradiation, electron-hole pairs are generated by two different routes. Specifically, electrons from the N 2p level are excited to the TiO_2_ conduction band (CB) by visible light, while those from the TiO_2_ valence band (VB) may be excited to the CB of the semiconductor by UV irradiation.

According to Wu et al. (2021) [10], the photocatalytic reduction of CO_2_ in the gas phase with N-doped TiO_2_ nanoparticles begins with the adsorption of CO_2_ molecules on the surface of the catalyst to form carbonate species. Then, electrons produced by the photocatalytic mechanism described above may reduce these adsorbed CO_2_ molecules to product CO through the protonation of ·COOH intermediate. Density functional theory (DFT) calculations found that enhanced surface polarization, due to N doping and oxygen vacancy, gives rise to significant charge accumulation on CO_2_ molecules, leading to the activation of CO_2_, which reduces the energy barrier to generate intermediate products and facilitate electron transfer at the interface [8]. In the case of CH_4_, the proposed mechanism implies the reaction of adsorbed HCO_3_^−^ with an electron to form C· radicals, which can convert into CH_4_ after successive reactions with H· radicals, via CH_3_· radical intermediate [2].

In the case of N-doped TiO_2_/carbon support nanocomposites (Figure 2b), as some studies [13,31] have hypothesized, under simulated sunlight radiation electrons and holes may be generated and transferred between the interface of support and N-doped TiO_2_, leading to charge recombination possibly being effectively retarded in the N/TiO_2_/CNT and N/TiO_2_/rGO composites. The holes formed in the VB of N-doped TiO_2_ may oxidize H_2_O molecules absorbed on the surface of particles to generate O_2_ and protons. The photogenerated electrons could be transferred from the CB of N-doped TiO_2_ to the carbon support via a percolation mechanism [14], where they could reduce CO_2_ molecules to CO and methane [31]. The absence of CH_4_ in some supported catalysts (as in our N-doped TiO_2_/CNT and TiO_2_/rGO composites) suggests that the protons from water may fail to capture the photogenerated electrons to form H· radicals, because they fall into electron-rich aromatic cycles of support, where they could be stabilized, as it is hard for them to participate in the production of CH_4_. For this reason, in the case of unsupported N-doped TiO_2_, due to the absence of the conjugated aromatic system, H^+^ or H· radicals generated in the photocatalytic reaction may quickly be consumed by CO_2_ in the photocatalytic process, and CH_4_ and CO simultaneously detected [31].

To sum up, N-doping reduces the energy necessary to reduce CO_2_ into CO and CH_4_ (i.e., it enhances the light absorption of photocatalysts in the visible region), whereas carbon support enlarges the time required for charge recombination. Both measures lead to higher photocatalytic activity of the synthesized catalysts. Regarding selectivity towards CH_4_, it seems to be influenced by the availability of H· radicals coming from H_2_O oxidation.

### 3.3. Surface Morphology Analysis (TEM)

**Figure 3:**
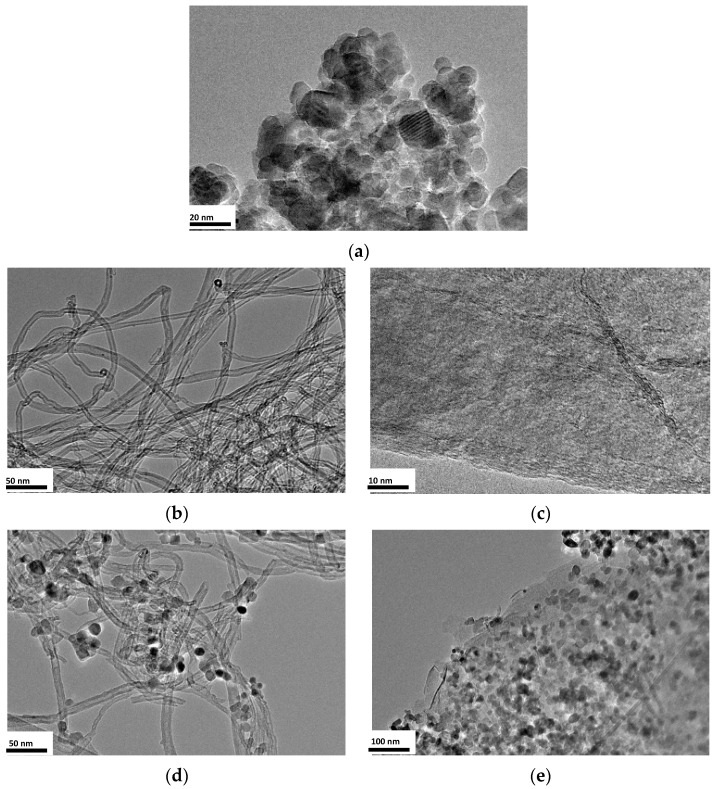
TEM images of different synthesized materials: (**a**) N^1^/TiO_2_, (**b**) N^2^/CNT, (**c**) N^10^/rGO, (**d**) N^1^/TiO_2_/CNT, (**e**) N^1^/TiO_2_/rGO.

TEM was carried out to analyze the structure and morphology of the samples and the results are shown in Figure 3.

In the case of N-doped TiO_2_ nanoparticles (Figure 3a), aggregates of polyhedral particles with crystallite sizes in the range of 11–14 nm and well-defined lattice fringes, suggesting a highly crystallized anatase structure, are observed [10]. The morphology is similar to undoped TiO_2_ [24], except that the crystallite sizes are larger [2]. Lucky and Charpentier (2010) observed that alkylamines used as N dopant in supercritical synthesis can form amine complexes with metal alkoxides, thus favoring the aggregation of the metal oxide particles [20]. All these findings will be corroborated by the results obtained in the next sections.

Regarding the supports (Figure 3b,c), neither N-doped CNT nor N-doped rGO show any difference from undoped supports [8,9]. When these supports are mixed with titania precursor, hydrolysis agent and N precursor in supercritical media, it is evident that N-doped TiO_2_ nanoparticles are successfully deposited on both CNT and rGO (Figure 3d,e).

Figure 3d shows that TiO_2_ nanoparticles are uniformly dispersed over CNT, as was found in previous works on N-doped TiO_2_/CNT composites synthesized with traditional synthesis [32] and undoped TiO_2_/CNT composites synthesized with supercritical fluids [8]. The crystallite size was about 10 nm, showing a narrower distribution than unsupported N-doped TiO_2_ nanoparticles (14 nm). In this sense, some works [33] explain that nitrogen-containing groups in the carbon support may serve as favorable nucleation and anchor sites for TiO_2_ nanocrystals. The smaller size of TiO_2_ nanoparticles in composites might be due to stronger coupling between TiO_2_ and N-doped sites on the support [33].

When TiO_2_/rGO composites are analyzed (Figure 3e), there is no sign of agglomeration of TiO_2_ nanoparticles, which are well distributed over the rGO support. The crystallite size of these particles is about 13–14 nm, like those of undoped TiO_2_/rGO composites synthesized with supercritical fluids (13 nm) [9]. Daraee et al. (2020) reached similar results when performing traditional synthesis [34].

If the influence of TiO_2_ crystallite size on photocatalytic activity is analyzed, it can be observed that photocatalytic activity may be directly related to crystallite size, since N-doped TiO_2_ nanoparticles and N-doped TiO_2_/rGO composites lead to higher CO_2_ reduction rates than N-doped TiO_2_/CNT composites. This phenomenon can be derived from a higher charge transfer, as will be shown in the corresponding section [9].

Before concluding this section, we should note that the photocatalysts N^1^/TiO_2_, N^1^/TiO_2_/CNT and N^1^/TiO_2_/rGO presented in this and the following sections were synthesized using 1 mL of TEA. This was done because, according to the results presented in Table 2, the N load in N-doped TiO_2_ nanoparticles and in N-doped TiO_2_ supported on CNT and rGO was always the same, regardless of the amount of TEA (N precursor) used in the synthesis (Table 2).

### 3.4. Crystalline Structure Analysis (XRD)

**Figure 4:**
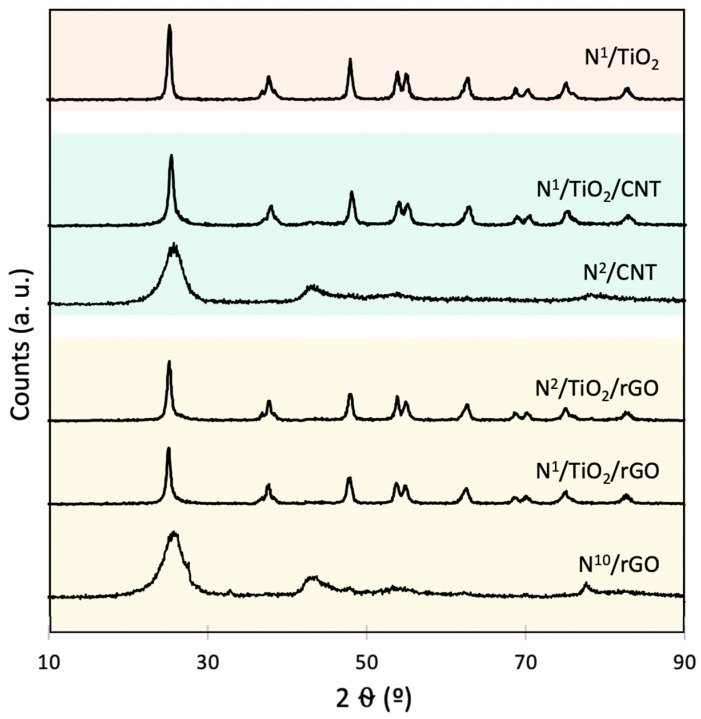
XRD diffractograms of different synthesized N-doped photocatalysts and supports.

The crystal structure and phase identification of TiO_2_ in the synthesized catalysts were investigated by using XRD technique. The XRD diffractograms are displayed in Figure 4.

In all samples, no matter if they were bare TiO_2_ [24] or supported on CNT [8] or rGO [9], the patterns were well matched with anatase-phase TiO_2_, indicating that the crystalline structure of synthesized TiO_2_ was not affected by doping [2] and supporting during the one-pot synthesis process [34,35].

Changes were observed in the peak shape and intensity in XRD patterns of N-doped TiO_2_ particles with respect to that of undoped TiO_2_ [36]. The increase in peak intensity of N-doped TiO_2_ catalysts, compared to that of undoped TiO_2_, indicates that N doping could enhance the crystallinity of TiO_2_ particles [2].

The crystallite sizes of modified TiO_2_ catalysts were estimated from XRD patterns using the Scherrer equation and are listed in Table 4. All synthesized catalysts possess smaller crystallite sizes than the reference P25 (20 nm) [37]. In our case, the crystallite sizes of N-doped TiO_2_ nanoparticles increased from 11 to 14 nm with increasing amounts of nitrogen from 0 to 2 mg N/g TiO_2_. This trend was observed in N-doped TiO_2_ catalysts obtained with traditional methods [37]. The values agree with those reported in a former study dealing with the synthesis of N-doped TiO_2_ catalysts in supercritical fluids [20]. It can be concluded that the presence of nitrogen doping influences the crystallite size of TiO_2_ grown during the doping process [30], as was observed in TEM images.

No drastic shift or presence of new peaks were observed, indicating that N doping did not lead to the formation of any secondary and impurity phases in the host TiO_2_, rather than occupancy of oxygen sites or inclusion in TiO_2_ lattice [5], which will be discerned later.

Regarding composites with CNT, the peaks in N-doped composites were wider than those in the undoped CNT-supported composite [8], which shows that degree of crystallization of TiO_2_ is slightly weakened by ion implantation [38], contrary to what happened with unsupported catalysts. In this sense, crystallite size decreased from 16 to 10 nm when undoped TiO_2_/CNT composites were doped with 2 mg N/g TiO_2_. This finding could be related to stronger coupling between TiO_2_ and N-doped sites on the support, as was explained in the previous section [33]. No characteristic peaks of CNTs were found in the composites, which may be the result of overlap between the intense peaks of CNTs and anatase at 25.9° and 25.2°, respectively [35]. This could also be attributed to the homogeneous coverage of TiO_2_ on CNTs [8].

In the case of composites with rGO, it can be observed that peaks of N-doped TiO_2_/rGO composites were slightly narrower than those of undoped TiO_2_/rGO composites [9]. This is probably due to altering the crystallite size of base TiO_2_ crystallites [34]. As a result, a small increase in crystallite size, up to 14 nm, was observed upon 1 mg N/g TiO_2_ doping. When N content increased (as in N^2^/TiO_2_/rGO), crystallite size decreased. Just as happened with CNTs, the main characteristic peak of graphene at about 25° is shadowed by the main peak of anatase TiO_2_, surely due to the homogeneous dispersion of TiO_2_ on rGO [39].

To sum up, measurements of crystallize sizes agreed with the results observed by TEM methodology and supported the trends in photocatalytic activity presented in Section 3.2.

### 3.5. Surface Area Analysis (BET)

Specific surface area is another critical parameter in determining the photocatalytic activity of TiO_2_. If a catalyst exhibits large surface area, the adsorption of many molecules takes place on its surface and reactions are promoted [37]. However, a large surface area is generally related to more crystalline defects. An excess of defects could assist in recombination processes of charge carriers and induce poor photocatalytic activity. Thus, an adequate surface area is a prerequisite, but not a deciding factor for a higher activity [40].

We observed that all isotherms of bare and N-doped TiO_2_ nanoparticles (depicted in Figure S1 in Supplementary Materials) displayed the typical structure of type IV isotherms with well-defined H1 hysteresis loops, indicating the characteristic of capillary condensation within uniform mesoporous structures, and confirming that mesoporous structures were well retained in TiO_2_ nanoparticles during the simultaneous processes of synthesis and nitrogen doping [2].

Table 4 presents the BET areas of the synthesized materials. The values for N-doped TiO_2_ nanoparticles agree with those reported by Lucky and Charpentier (2010) when this type of catalyst was obtained in supercritical medium [20]. As shown, in the presence of N a decrease in the specific surface area was observed [2]. This could be attributed to the higher crystallite sizes of N-doped TiO_2_ catalysts described in the previous section and caused by N present in the form of interstitial N (Ti-O-N or Ti-N-O), or substitutional N (Ti-N), since the N^3−^ ion has a larger ionic radius (0.171 nm) than the O^2−^ ion (0.140 nm) [2]. The presence and abundance of these N species will be treated more deeply in Section 3.7.

The supported catalysts exhibit similar or larger specific surface areas than TiO_2_ nanoparticles due to the presence of carbon supports. The values coincide with those of composites obtained by both traditional [34,41] and high-pressure methods [8,9]. Moreover, there was reduction in support surface area in the TiO_2_/support composites that suggests the existence of a partial blockage of CNTs inner surface [8] and partial rGO surface coverage [9].

Finally, it can be verified that opposite trends of crystallite sizes and BET areas were fully met in our experimental results, both for unsupported and supported TiO_2_ catalysts (Table 4).

### 3.6. Surface Functional Groups Analysis (FTIR)

**Figure 5:**
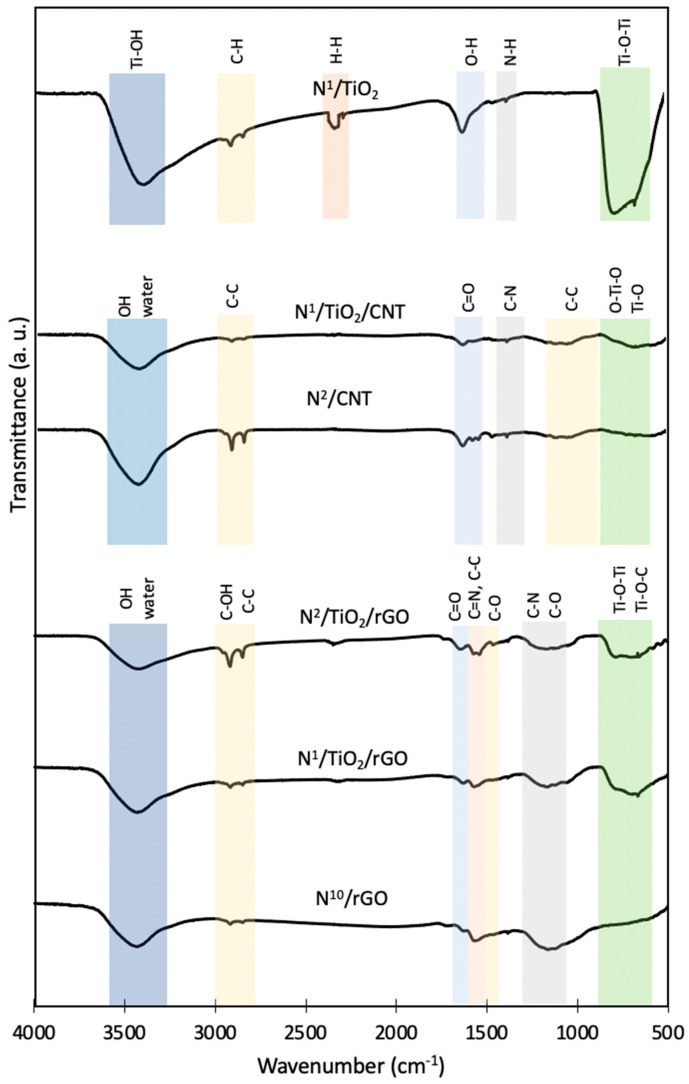
FTIR spectra of different synthesized N–doped TiO_2_–based photocatalysts and supports.

The FTIR spectra of the synthesized materials are shown in Figure 5. In the case of N-doped TiO_2_ nanoparticles, the broad band in the region 3600–3200 cm^−1^ can be ascribed to the stretching vibration of the surface-bonded Ti-OH groups, which may act as proton source to decrease CO_2_ activation energy during the reduction process [10]. Moreover, this band broadens and shifts to a lower wavenumber in N-doped TiO_2_ nanoparticles in contrast to undoped TiO_2_, due to the incorporation of N atoms and N-containing groups into TiO_2_ [42]. The weak bands at about 2900 cm^−1^ can be related to the stretching-vibration mode of C-H bonds that could possibly derive from the residues produced during the calcination of the precursors involved [5]. The small peak at around 2340 cm^−1^ can be associated to the bending vibration modes of the H-H bond, and the peak around 1630 cm^−1^ to the bending vibration of O-H of the physisorbed water molecules [5]. The band around 750 cm^−1^ is assigned to the characteristic stretching-vibration mode of Ti-O-Ti bonds of anatase TiO_2_. This peak is sharper and suffers from a shifting to higher wavenumber in N-doped TiO_2_ because of the O-Ti-N and N-Ti-N linkage [8,40]. The tiny peak around 1375 cm^−1^ could correspond to trace N atoms (N-H linkage) that are substituted into the lattices of TiO_2_, or be due to the presence of molecular residues from triethylamine [5,42].

Regarding N-doped CNT and TiO_2_/CNT, the presence of OH groups and water on the surface of the catalysts was confirmed by the appearance of a broad band at about 3400 cm^−1^ [13]. As explained before, the presence of hydroxyl groups on the composite surfaces plays an important role in photocatalytic activity. The band due to the stretching and bending modes of Ti-O and O-Ti-O appears as a broad band at about 600 cm^−1^ in the spectra of the composites [13]. Some characteristic peaks of CNT are observed in composites due to the large percentage of CNT in the nanocomposites, such as peaks in the region 2980–2880 and 1000 cm^−1^ (C-C bonds), and at about 1600 cm^−1^ (carbonyl C=O bonds). The weakening of the intensity of the peaks in the nanocomposites is due to the breaking down of CNT walls to its graphitic fragments and the attachment of these graphitic fragments onto, and into, the TiO_2_ nanocrystals [42]. This confirms the incorporation of CNT into the nanocomposites. The incorporation of N into carbon material was also demonstrated by the small C-N peak at 1325 cm^−1^ [42].

In addition to the striking peak at 3400 cm^−1^ related to OH groups and mentioned in the two previous cases, N-doped rGO and TiO_2_/rGO composites exhibited signals at 2800, 1625 and 1500 cm^−1^ associated with the stretching of C-OH, the presence of C=O and the deformation of C-O groups of rGO [43]. The 2800 cm^−1^ signal could overlap with C-C signals at 2852 and 2919 cm^−1^ [44]. These surface oxygen-containing functional groups render the possibility of covalent linkage of TiO_2_ onto the rGO surface [45]. The main difference between N-doped TiO_2_/rGO composites and rGO is the band at 650 cm^−1^ related to the Ti-O-Ti bonds. The broadening of this peak may suggest the presence of a peak due to Ti-O-C bond. This confirms that TiO_2_ nanoparticles could be strongly bonded to graphene sheets [34]. In addition, the peaks at about 1360 and 1550 cm^−1^ showed the possible presence of C-N bond from pyrrolic nitrogen (interstitial) and C=N from pyridinic nitrogen (substitutional), respectively [46]. Although they can also be related to C-O and C-C bond bands, respectively [47]. N^2^/TiO_2_/rGO catalyst does not show any difference with the composite with lower N content.

### 3.7. Surface Chemical Analysis (XPS)

**Figure 6:**
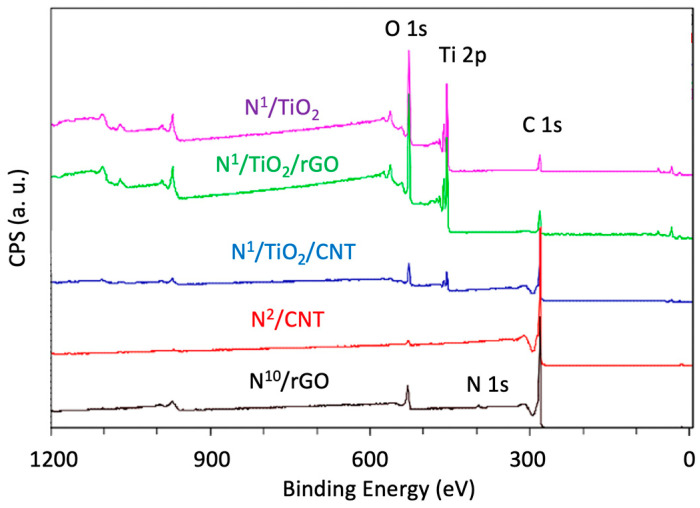
Full scan XPS spectra of some synthesized N-doped TiO_2_-based photocatalysts and supports.

The chemical state and surface composition of N-doped catalysts were investigated using the XPS technique. The full scan spectra are displayed in Figure 6 and show the existence of N, O, Ti and C in the samples. In the case of N-doped TiO_2_ nanoparticles, C 1s peak can be ascribed to remnant organic precursors not completely removed during the calcination [37].

**Figure 7:**
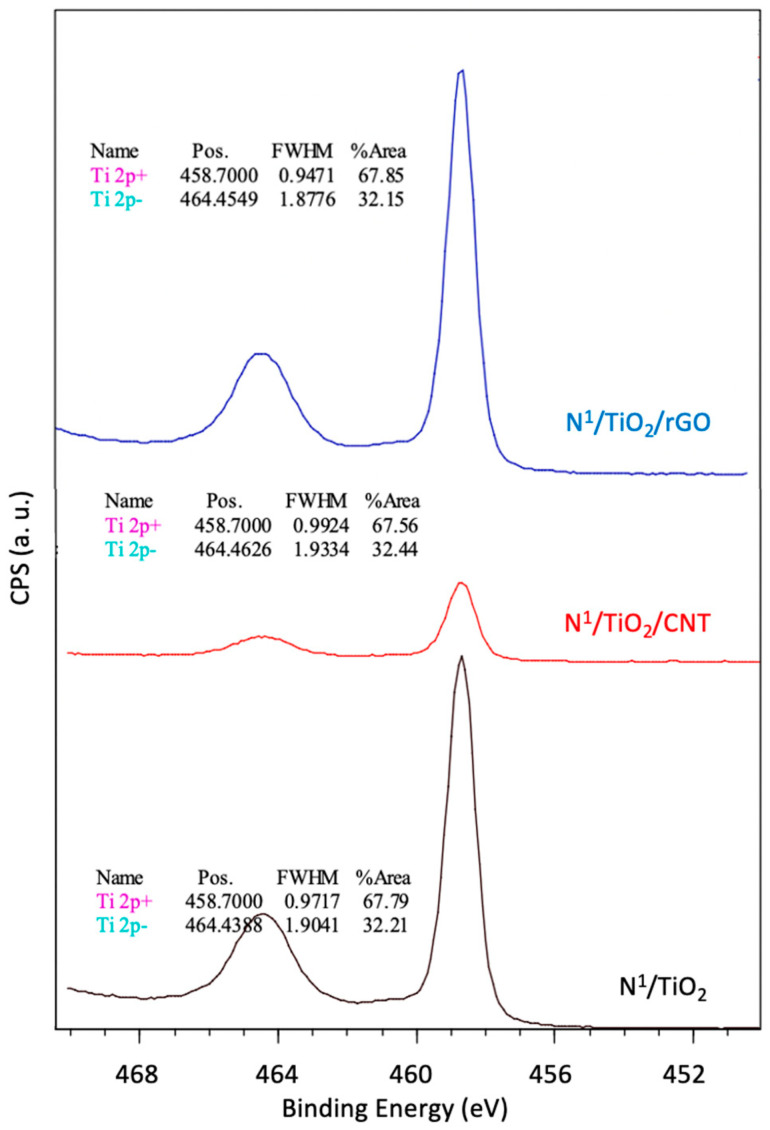
Narrow scan XPS spectra of Ti 2p of different synthesized N–doped TiO_2_–based photocatalysts.

The narrow scan Ti 2p spectra of N-doped TiO_2_, TiO_2_/CNT and TiO_2_/rGO (Figure 7) identified two Ti characteristic peaks located at 458.5 and 464.2 eV. They correspond to typical binding energies of Ti^4+^ (Ti 2p_3/2_ and Ti 2p_1/2_ of TiO_2_) [48]. However, these peaks were 0.65 eV lower than those of bare TiO_2_, which may be an indication of successful N doping. The N element is less electronegative than the O element. When N atoms are present in the TiO_2_ lattice, a part of Ti^4+^ is reduced to Ti^3+^, which may lead to decrease in the binding energy of Ti 2p [38]. The absence of other non-Ti^4+^ species or deconvoluted peaks of Ti could also be due to the resolution of XPS, which was unable to detect minor changes of TiO_2_ or because the Ti^3+^ species exist in the subsurface or bulk, which is inaccessible by XPS [37]. In any case, according to our results it seems that the replacement of oxygen atoms with nitrogen atoms in the TiO_2_ structure of both TiO_2_ nanoparticles and nanocomposites may have not occurred [5]. To sum up, Figure 7 depicts that TiO_2_ is present in all catalysts, and this TiO_2_ may be interstitially doped with N.

**Figure 8:**
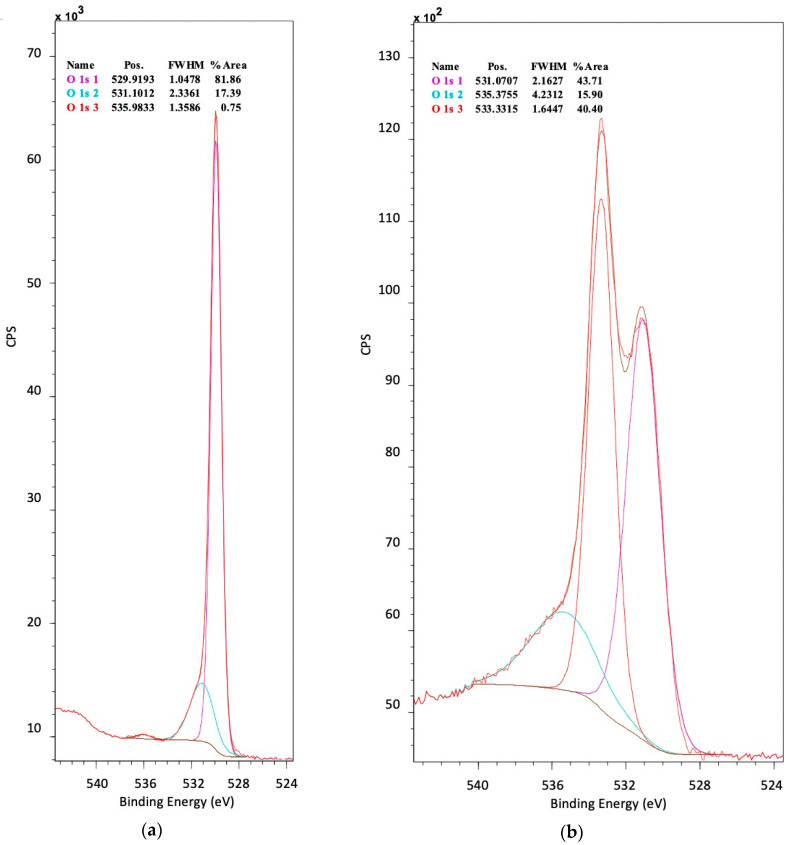
Narrow scan XPS spectra of O 1s of different synthesized materials: (**a**) N^4^/TiO_2_/rGO, (**b**) N^10^/rGO.

The deconvoluted O 1s spectra of N-doped TiO_2_ nanoparticles, TiO_2_/CNT and TiO_2_/rGO composites (the latter as example, Figure 8a) showed the peak at 529 eV representing the stoichiometric existence of oxygen network in TiO_2_ with respect to Ti (Ti-O) as well as the doped N (Ti-O-N) [5]. The peaks at 531 eV and 535 eV corresponded to surface adsorbed oxygen and water, respectively [49,50]. When O 1s spectra of supports and composites were compared (N^10^/rGO as example, Figure 8b), new peaks at 531 and 533 eV appeared, related to C=O (carbonyl, carboxyl) and C-O (epoxy, hydroxyl) groups, respectively [50]. All these results imply that TiO_2_ may be doped with N, and the existence of functional groups in supports are susceptible to have bound TiO_2_ particles.

**Figure 9:**
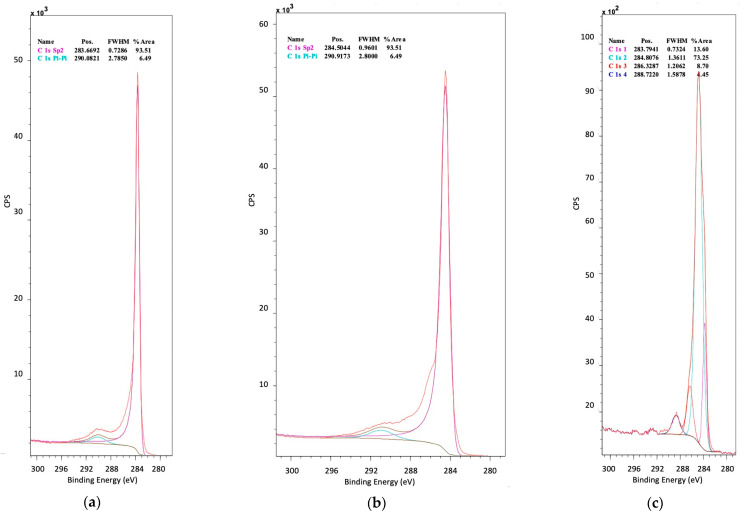
Narrow scan XPS spectra of C 1s of different synthesized photocatalysts and supports: (**a**) N^1^/TiO_2_/CNT, (**b**) N^10^/rGO, (**c**) N^1^/TiO_2_/rGO.

In the case of N-doped CNT and TiO_2_/CNT composites (shown N^1^/TiO_2_/CNT as example in Figure 9a), the C 1s spectra are deconvoluted into two peaks at 283.6–284.5 and 290.1–290.9 eV [17]. The first one is bigger and due to graphitic carbon in CNT, whereas the second one is related to C=O/C-N bonds [50]. C 1s spectra of N-doped rGO (Figure 9b) and TiO_2_/rGO composites (Figure 9c) can be deconvoluted into 2 and 4 peaks, respectively. The peaks at 283.8–284.5 eV and 290.9 eV indicate the presence of graphene C-(C,H) and O-C-O bonds in rGO, respectively [43]. The relatively weak signal of the C-O groups indicates that most of the GO oxygen is reduced during the synthesis of catalysts in supercritical medium [51]. The first peak also appears in the N-doped TiO_2_/rGO composite, proving that the structure of graphene remains after the synthesis of composite catalyst [39]. Moreover, the two small peaks at 286.3 and 288.7 eV in TiO_2_/rGO composite can be associated with C-O and C=O bonds in support, respectively.

**Figure 10:**
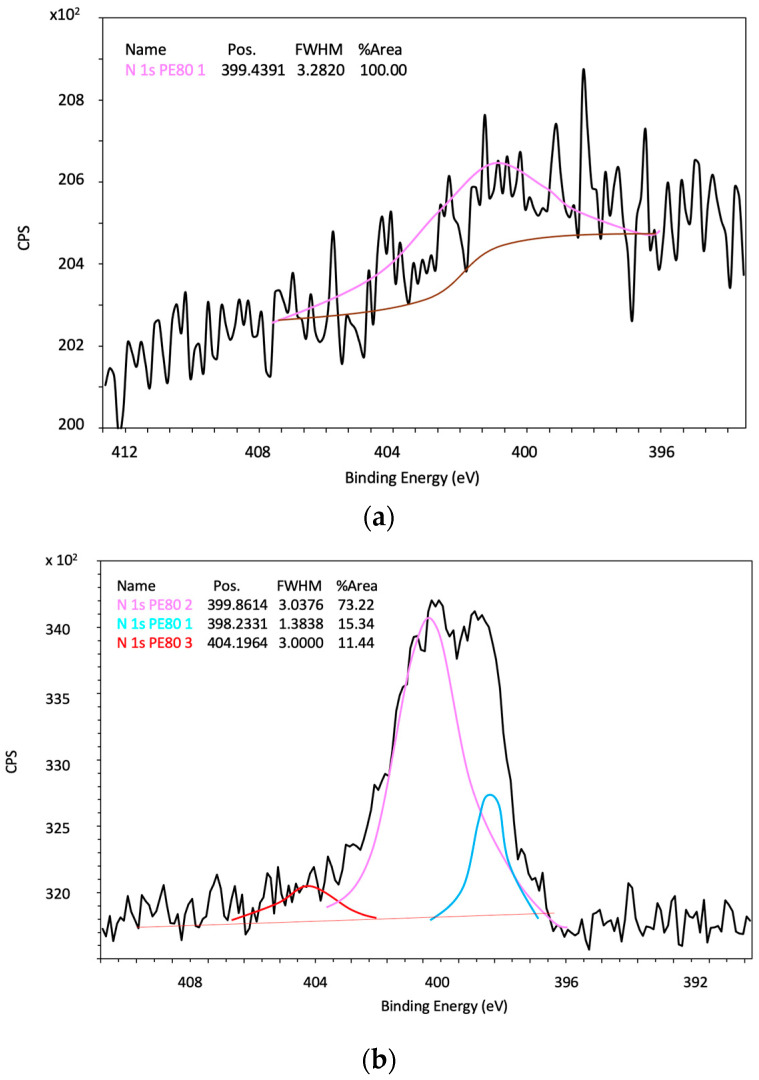
Narrow scan XPS spectra of N 1s of different synthesized photocatalysts and supports: (**a**) N^1^/TiO_2_/CNT, (**b**) N^10^/rGO.

In N-doped TiO_2_ nanoparticles, CNT, rGO, TiO_2_/CNT and TiO_2_/rGO composites (N^1^/TiO_2_/CNT for example, Figure 10a), the only peak at 399.4-399.8 eV corresponding to N 1s confirms the existence of interstitial N in TiO_2_ (Ti-O-N) and the absence of substitutional N (N-Ti-N) [5]. According to Wang et al. (2009), at relatively low calcination temperature (<600 °C), N atoms tend to sit in the interstitial sites, above all if the N atomic percentage is below 1.2 [52]. At a relatively high calcination temperature (600 °C), some of the N atoms are incorporated into the TiO_2_ lattice substitutionally, in addition to the presence of interstitial N atoms. In the case of N-doped rGO (Figure 10b) two additional peaks at 398.2 and 404.2 eV are present. The first one can be related to pyridinic N, whereas the second one to C-N-O, indicating the successful doping of N atoms into the graphene framework [51].

From XPS analysis, the atomic percentage of N in the TiO_2_ crystal lattice is about 0.22 in N-doped TiO_2_ nanoparticles and N-doped TiO_2_/rGO composite, but 0.13 in N-doped TiO_2_/CNT composite. There seems to be some kind of correlation with the support used, since N-doped CNT and rGO exhibit 0.06 and 1.06 atomic percentage of N, respectively. If these figures are compared with those obtained in Section 3.1 with elemental analysis, it seems that N incorporation takes place mainly on the surface in the case of TiO_2_ nanoparticles and TiO_2_/rGO composites, but in the bulk in the case of bare CNT and TiO_2_/CNT nanocomposites [27]. This could also have contributed to the lower photocatalytic yield of N-doped TiO_2_/CNT composites, where N-containing active sites are less accessible.

As a summary, XPS analyses allow us to state that the one-pot supercritical process achieved interstitial N-doping within both the TiO_2_ lattice structure and carbon support framework, almost complete reduction of GO into rGO, and the preservation of the graphitic structure of the supports.

### 3.8. Optical Properties Analysis (DRS)

**Figure 11:**
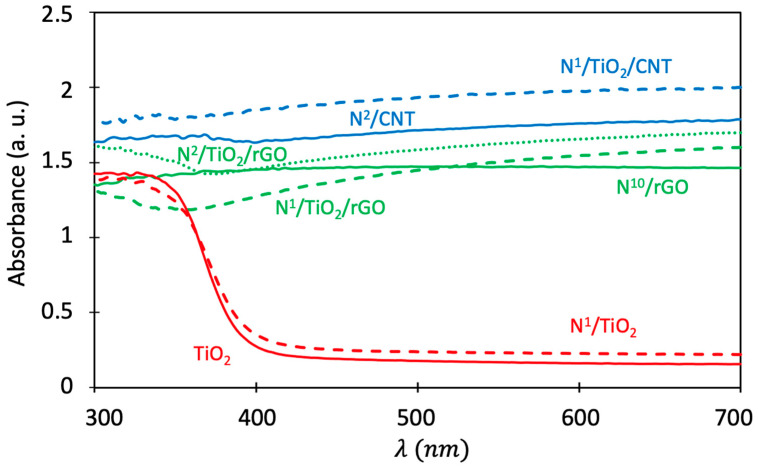
DRS spectra of different synthesized N-doped TiO_2_-based photocatalysts and supports.

The optical UV-vis light absorption characteristics of the synthesized catalysts were investigated using diffuse reflectance UV-visible absorption spectroscopy. Some of the obtained spectra are displayed in Figure 11. The band gap energy and absorption threshold of the synthesized samples and supports were estimated as in previous works [6] and are given in Table 4.

On the one hand, it can be seen in the absorption spectra that the absorption threshold of undoped TiO_2_ nanoparticles at 400 nm was shifted to 405 nm in the case of TiO_2_ nanoparticles with 1 mg N/g TiO_2_. This indicates that N-doping slightly expanded the optical absorption of TiO_2_ nanoparticles to the visible light region. Accordingly, the band gap energy of undoped and N-doped TiO_2_ nanoparticles were 3.10 and 3.06 eV, respectively. This enhancement in optical properties could result from the formation of energy levels near and above the valence band (VB) of TiO_2_ due to doped N atoms [5]. This slight decrease in band gap energy agrees with works on the traditional synthesis of N-doped TiO_2_ [2].

As explained before, in the doping procedure N can create space for itself in the bulk or on the surface. If the crystallization of titania occurs while the dopant source is added, the N incorporates in the crystal lattice [53]. The dopant species could be incorporated in the crystal lattice occupying either a substitutional (Ti-N) or an interstitial site (Ti-O-N), which leads to the formation of a new band between the CB and VB of titania, resulting in reduction of the band gap energy [53]. Substitutional doping involves oxygen replacement, whereas interstitial doping involves the addition of nitrogen into TiO_2_ lattices. Substitutional N introduces localized nitrogen states up to 0.14 eV above the VB and interstitial N forms Π-character states up to 0.74 eV above the VB. The excitation from the occupied high energy levels to the CB is more favorable with interstitial N-doped TiO_2_, exhibiting higher visible light activity [53]. However, the absorbance of a photocatalyst cannot be directly correlated to its photoactivity, so improvement in photocatalytic activity may not necessarily be observed, due to band gap reduction [40].

Regarding the influence of calcination temperature on light absorption, Sathish et al. (2005) found that the light absorption of N-TiO_2_ particles in the visible region decreased very significantly as the calcination temperature increased above 400 °C, due to a decrease in the amount of N doping in TiO_2_ with calcination temperature [54].

On the other hand, it is not surprising that the light absorption spectra of composites and supports (CNT or rGO) are similar since the composite surface is not fully covered with TiO_2_. The particular shape of absorbance curves for carbon supports and composites has also been observed in other works dealing with traditional synthesis of N-doped TiO_2_/carbon support composites [39]. Precisely, this very special form prevents us from calculating the band gap energies of composites with the same graphical method described in reference [6], as happened in previous works [8,9]. For this reason, it is necessary to apply Tauc’s graphical procedure (Figure S2) [15]. With it, values of band gap energies of about 2.10 eV and 2.40 eV were obtained for N/TiO_2_/CNT and N/TiO_2_/rGO, respectively, proving that the composites have higher visible light absorbance after N-doping and loading of N-TiO_2_ [55]. In the case of the TiO_2_/rGO catalyst, with higher N content (N^2^/TiO_2_/rGO), the absorbance was slightly higher than that of N^1^/TiO_2_/rGO, being the value of the band gap energy 0.05 eV smaller.

These findings may be due to doping of N into TiO_2_ lattice narrows its band gap whilst the carbon support decoration could also improve photo-absorption in the visible light region and reduce the reflection of light [55]. This shift in the absorption threshold to the visible light range is consistent with the color change observed in powders, from white (undoped TiO_2_) to light grey (N-doped TiO_2_) and dark grey/black (N-doped TiO_2_/CNT and N-doped TiO_2_/rGO) [56].

### 3.9. Electrical Properties Analysis

**Figure 12:**
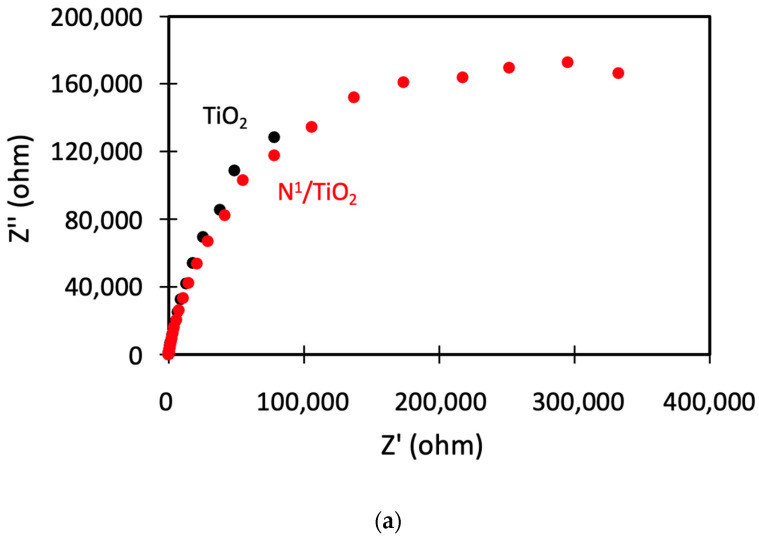

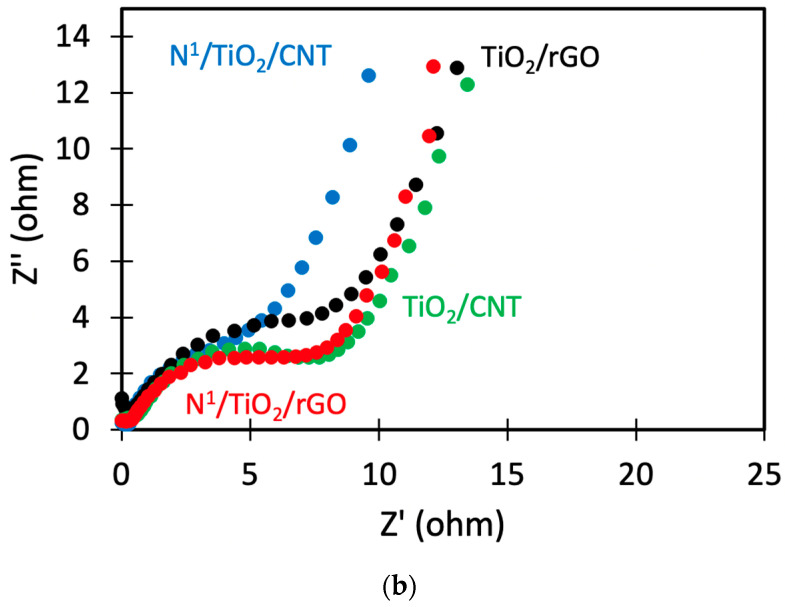
Electrochemical impedance spectra: (**a**) TiO_2_ nanoparticles and N-doped TiO_2_ nanoparticles, (**b**) TiO_2_/CNT, N-doped TiO_2_/CNT, TiO_2_/rGO and N-doped TiO_2_/rGO composites.

Finally, electrochemical impedance spectroscopy (EIS) was employed to evaluate the photo-excited charge-transfer property of the photocatalysts. Nyquist plots (Z″ vs. Z′) of the different photocatalysts are depicted in Figure 12.

Undoped TiO_2_ and N-doped TiO_2_ nanoparticles (N^1^/TiO_2_ as example) show a similar semicircular shape (Figure 12a). As the arc radii are alike, this implies similar resistance for charge transfer and similar charge separation efficiency for both photocatalysts. The results are coherent with those corresponding to TiO_2_ nanoparticles synthesized with traditional methods [57] and it is expected that the arc radius would be reduced if far more N content could be introduced into the photocatalyst [10].

In the case of CNT/TiO_2_ and rGO/TiO_2_ composites, all four catalysts show the typical characteristics of one semicircle in the middle-high frequency range and a sloping straight line in the low frequency (Figure 12b). The arc radii of the EIS Nyquist plot of the composites are far smaller than those of TiO_2_ nanoparticles [33,51], indicating that the interface layer resistance and the charge transfer resistance on the surface are diminished, which reveals that charge migration is facilitated by interfacial interaction between the TiO_2_ and carbon material (CNT or rGO) occurring in the TiO_2_-C heterojunction [58].

Regarding N-doping, EIS Nyquist plots show that the arc radius for N^1^/TiO_2_/rGO is noticeably smaller than the undoped TiO_2_/rGO composite (TiO_2_/rGO weight ratio equal to unity). This is due to the presence of N in both TiO_2_ nanoparticles [10] and carbon support [59].

On the contrary, N^1^/TiO_2_/CNT nanocomposite exhibits larger arc radius (higher resistance for charge transfer and lower charge separation efficiency) than the undoped TiO_2_/CNT composite. This behavior has already been explained in previous sections in terms of smaller crystallite size and may be due to the presence of N in the bulk but not on the surface of the N-doped TiO_2_/CNT composite.

Moreover, the order of increasing the arc radius almost fully matches with the order of catalysts according to their decreasing photocatalytic activity: N^1^/TiO_2_/rGO = TiO_2_/CNT > TiO_2_/rGO > N^1^/TiO_2_ > N^1^/TiO_2_/CNT > TiO_2_.

### 3.10. Summary of Properties

In this section, the different properties exhibited by the N-doped catalysts synthesized in supercritical medium will be summarized and compared with those of catalysts synthesized by traditional methods, as well as those obtained with supercritical fluids but not doped with N.

Generally speaking, N-doped TiO_2_ nanoparticles obtained with supercritical fluids in this work exhibited an improved photocatalytic activity in terms of both total conversion and methane selectivity than those obtained with traditional methods [29]. This enhanced behavior seems to be derived from a lower degree of aggregation [2], larger crystallite size [2] and slightly higher visible light absorption [2]. Regarding N-doped TiO_2_/carbon support composites synthesized in supercritical medium, only N-doped TiO_2_/rGO composites have shown higher photocatalytic activity (but not methane selectivity) than similar composites synthesized with traditional methods [30]. In this case, the main reason is undoubtedly the extraordinarily good ability of the composites obtained in this work to absorb visible light compared to conventional N-doped TiO_2_/rGO materials [39]. The poorer photocatalytic activity of N-doped TiO_2_/CNT composites seems to be derived from the smaller crystallite size [32] and BET area [34] of the materials obtained in this work, in contrast to those synthesized with traditional methods.

In the case of the photocatalysts obtained in a supercritical medium by our group in previous studies, the main advantage of the materials described in this work is that N-doping allowed the photocatalytic activity shown by metal-doped catalysts to be maintained, with consequent saving of more expensive raw materials (Cu, Pd, Pt). Even in the case of N-doped TiO_2_ nanoparticles, methane selectivity was doubled in contrast to undoped [36] and metal-doped nanoparticles [60], probably due to the larger crystallite size of the first ones. Something similar was observed for N-doped TiO_2_/rGO composites, although methane selectivity of metal-doped TiO_2_/rGO composites was not improved [9]. N-doped TiO_2_/rGO composites exhibited higher BET area and lower band gap energy, but smaller crystallite size than metal-doped TiO_2_/rGO composites [9]. Finally, N-doped TiO_2_/CNT composites showed lower photocatalytic activity than undoped and metal-doped TiO_2_/CNT composites [8]. In this case, the small crystallite size seems to hinder its excellent properties related to visible light absorption [8]. Moreover, XPS analysis suggested that N incorporation took place mainly in the bulk, but not on the surface, in N-doped TiO_2_/CNT composites, as the N-containing active sites were less accessible [27].

## 4. Conclusions

N-doped TiO_2_ nanoparticles, N-doped TiO_2_/CNT and N-doped TiO_2_/rGO nanocomposites were synthesized by a facile one-pot method in a supercritical medium. The presence of N in both TiO_2_ and carbon supports endowed them with good visible light sensitization and high separation efficiency of the charges photogenerated after irradiation with solar light. The photocatalysts exhibited good photocatalytic performance in photoreduction of CO_2_ in the presence of water vapor and the highest conversion rate of 8 µmol/gTiO_2_/h was achieved with N^1^/TiO_2_/rGO composite. The photocatalytic products depended on the catalyst type. CO and CH_4_ were formed on N-doped TiO_2_ nanoparticles (CH_4_/CO ratio 2.5), while almost only CO was produced on both composites (N-doped TiO_2_ on CNT or rGO) as a result of a lack of H· radicals coming from H_2_O oxidation. The specific N content of the catalysts could be regulated by varying the N precursor, leading urea to higher N levels in the catalysts than TEA. Nevertheless, similar properties and even lower photocatalytic activity were exhibited by the composites with higher N percentage (N^2^/TiO_2_/rGO and N^2^/TiO_2_/CNT).

To sum up, in the present work the effect of the carbon support (CNT and rGO) on the activity and selectivity of the N-doped TiO_2_ nanoparticles in the CO_2_ photocatalytic reduction reaction was evaluated. Specifically, it was found that maximum CO_2_ conversion was achieved with the rGO support (N^1^/TiO_2_/rGO (b)). It almost doubled that obtained when using CNT (N^1^/TiO_2_/CNT (b)). However, no differences in selectivity were achieved with both carbon supports.

The results regarding N-doped nanoparticles and N-TiO_2_/rGO nanocomposites are of special interest, especially in terms of methane selectivity and total conversion, respectively. Nevertheless, as a promising avenue for future research, we may suggest the modification of N-doped TiO_2_-based photocatalysts investigated in this work with an additional metallic dopant. The interesting results reported in studies of charge generation and transfer conducted with metal-doped TiO_2_ support this hypothesis.

## Figures and Tables

**Table 1:** N content of the different N-doped supports.

Support	Nitrogen Precursor Used in the Synthesis	N Content (mg N/g Support)	Material Name ^(1)^
CNT	1 mL TEA 2 mL TEA 4 mL TEA 0.3 g urea	1.0 2.1 2.0 1.9	N^1^/CNT N^2^/CNT (a) N^2^/CNT (b) N^2^/CNT (c)
rGO	1 mL TEA 2 mL TEA 4 mL TEA 0.3 g urea	4.1 7.0 10.2 42.4	N^4^/rGO N^7^/rGO N^10^/rGO N^42^/rGO

^(1)^: N^X^/CNT or N^X^/rGO are materials names, where X is N content in mg N/g carbon support. (a), (b) and (c) are employed to differentiate materials with the same N content.

**Table 2:** N content of the different N-doped photocatalysts.

Photocatalyst	Nitrogen Precursor Used in the Synthesis	N Content (Measured by Elemental Analysis) (mg/g Photocatalyst)	N Content (Measured or Estimated) (mg N/g TiO_2_)	Photocatalyst Name ^(1)^
N-doped TiO_2_ nanoparticles	1 mL TEA 2 mL TEA 4 mL TEA 0.3 g urea	1.1 1.0 1.0 2.1	1.1 1.0 1.0 2.1	N^1^/TiO_2_ (a) N^1^/TiO_2_ (b) N^1^/TiO_2_ (c) N^2^/TiO_2_
N-doped TiO_2_ nanoparticles supported on CNT	1 mL TEA 2 mL TEA 4 mL TEA 0.3 g urea	1.0 1.4 1.6 2.1	1 1 1 2	N^1^/TiO_2_/CNT (a) N^1^/TiO_2_/CNT (b) N^1^/TiO_2_/CNT (c) N^2^/TiO_2_/CNT
N-doped TiO_2_ nanoparticles supported on rGO	1 mL TEA 2 mL TEA 4 mL TEA 0.3 g urea	2.6 4.0 5.6 22.2	1 1 1 2	N^1^/TiO_2_/rGO (a) N^1^/TiO_2_/rGO (b) N^1^/TiO_2_/rGO (c) N^2^/TiO_2_/rGO

^(1)^: N^X^/TiO_2_, N^X^/TiO_2_/CNT and N^X^/TiO_2_/rGO are material names, where X is N content in mg N/g TiO_2_. (a), (b) and (c) are employed to differentiate materials with the same N content.

**Table 3:** Photocatalytic production rates of CO and hydrocarbons with N-doped TiO_2_-based photocatalysts reported in the bibliography.

Catalyst	Products (µmol/h/g TiO_2_)	Conditions	Reference
N^1^/TiO_2_	CO: 1.5 CH_4_: 3.7 C_2_H_6_ + C_3_H_8_: 0 Total: 5.2	See Materials and methods	This work
5 wt. % N-TiO_2_ (liquid phase)	CO: 0 CH_4_: 0.1 C_2_H_6_ + C_3_H_8_: Tr Total: 0.1	6 fluorescent bulbs (400–800 nm), 13 W, continuous flow stirred slurry reactor, 250 mL, 10 mL/min, catalyst concentration 1 g/L, 6 h	[2]
N-TiO_2_ (gas phase)	CO: 0.1 CH_4_: 0.2 C_2_H_6_ + C_3_H_8_: Tr Total: 0.3	Continuous, catalyst 100–300 mg, CO_2_:H_2_O = 30:1, CO_2_ flow 0.3 mL/min, Xe lamp (315–600 nm), irradiation area 1.08 × 10^−3^ m^2^	[29]
N^1^/TiO_2_/CNT	CO: 3.9 CH_4_: 0.1 C_2_H_6_ + C_3_H_8_: 0 Total: 4	See Materials and methods	This work
N^1^/TiO_2_/rGO	CO: 7.5 CH_4_: 0.2 C_2_H_6_ + C_3_H_8_: 0 Total: 7.7	See Materials and methods	This work
N-TiO_2_/graphene (gas phase)	CO: 0 CH_4_: 0.4 C_2_H_6_ + C_3_H_8_: 0 Total: 0.4	Continuous gas flow reactor, visible light irradiation (15 W), 10 h, CO_2_ flow 5 mL/min	[30]
g-C_3_N_4_-N-TiO_2_ (gas phase)	CO: 4.8 CH_4_: 3.3 C_2_H_6_ + C_3_H_8_: 0 Total: 8.1	Gas-closed circulation system, 780 mL, 0.1 g catalyst, 300 W Xe arc lamp	[31]

Tr: Traces. The gray background represents the same type of catalysts.

**Table 4:** Results of the characterization of different N-doped TiO_2_-based photocatalysts and supports.

Samples	N Content (mg N/g TiO_2_)	TiO_2_ Crystallite Size (nm)	BET Area (m^2^/g)	Band Gap (eV)	Absorption Threshold (nm)
TiO_2_	0	11	152	3.10	400
N^1^/TiO_2_	1	14	82	3.06	405
N^2^/TiO_2_	2	14	81	3.06	405
CNT	0	-	216	-	-
N^1^/CNT	1 mg N/g CNT	-	284	-	-
N^2^/CNT	2 mg N/g CNT	-	280	-	-
N^1^/TiO_2_/CNT	1	10	145	2.12	-
N^2^/TiO_2_/CNT	2	10	140	2.10	-
rGO	0	-	163	-	-
N^4^/rGO	4 mg N/g rGO	-	173	-	-
N^10^/rGO	10 mg N/g rGO	-	171	-	-
N^1/^TiO_2_/rGO	1	14	85	2.45	-
N^2^/TiO_2_/rGO	2	13	84	2.40	-

The gray background represents the same type of materials.

## Data Availability

Not applicable.

## Supplementary Materials

The following supporting information can be downloaded at: https://www.mdpi.com/article/10.3390/nano12111793/s1, Figure S1: BET isotherms of some N-doped photocatalysts and supports; Figure S2: Tauc’s plots of N^1^/TiO_2_/CNT and N^1^/TiO_2_/rGO.
