# Supplementary material for: Photocatalytic Reduction of CO2 with N-Doped TiO2-Based Photocatalysts Obtained in One-Pot Supercritical Synthesis
Source: Nanomaterials (Basel). 2022 May 24;12(11):1793. doi: 10.3390/nano12111793 (PMC9182572; doi:10.3390/nano12111793)
Supplement: Supplementary file 1 [file nanomaterials-12-01793-s001.zip › nanomaterials-1726177-supplementary.pdf]

Supporting information

Photocatalytic Reduction of CO_2 with N-Doped TiO_2-Based Photocatalysts Obtained in One-Pot Supercritical Synthesis

Óscar R. Andrade, Verónica Rodríguez, Rafael Camarillo *, Fabiola Martínez, Carlos Jiménez and Jesusa Rincón

Department of Chemical Engineering, Faculty of Environmental Sciences and Biochemistry, University of Castilla-La Mancha, Av. Carlos III, s/n, 45071 Toledo, Spain; oscarramiro.andrade@uclm.es (Ó.R.A.); veronica.rodriguez@uclm.es (V.R.); fabiola.martinez@uclm.es (F.M.); carlos.jimenez@uclm.es (C.J.); jesusa.rincon@uclm.es (J.R.)

* Correspondence: rafael.camarillo@uclm.es

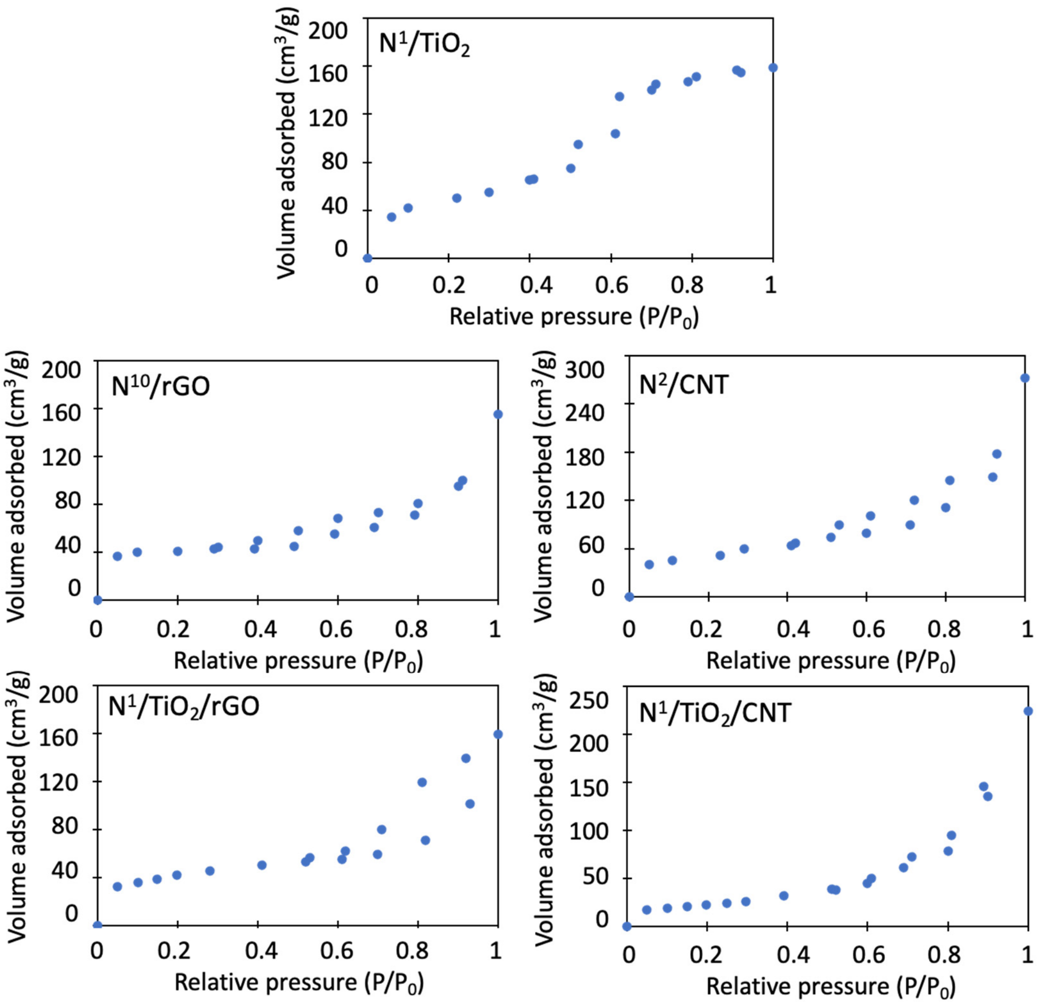

Figure S1. BET isotherms of some N-doped photocatalysts and supports.

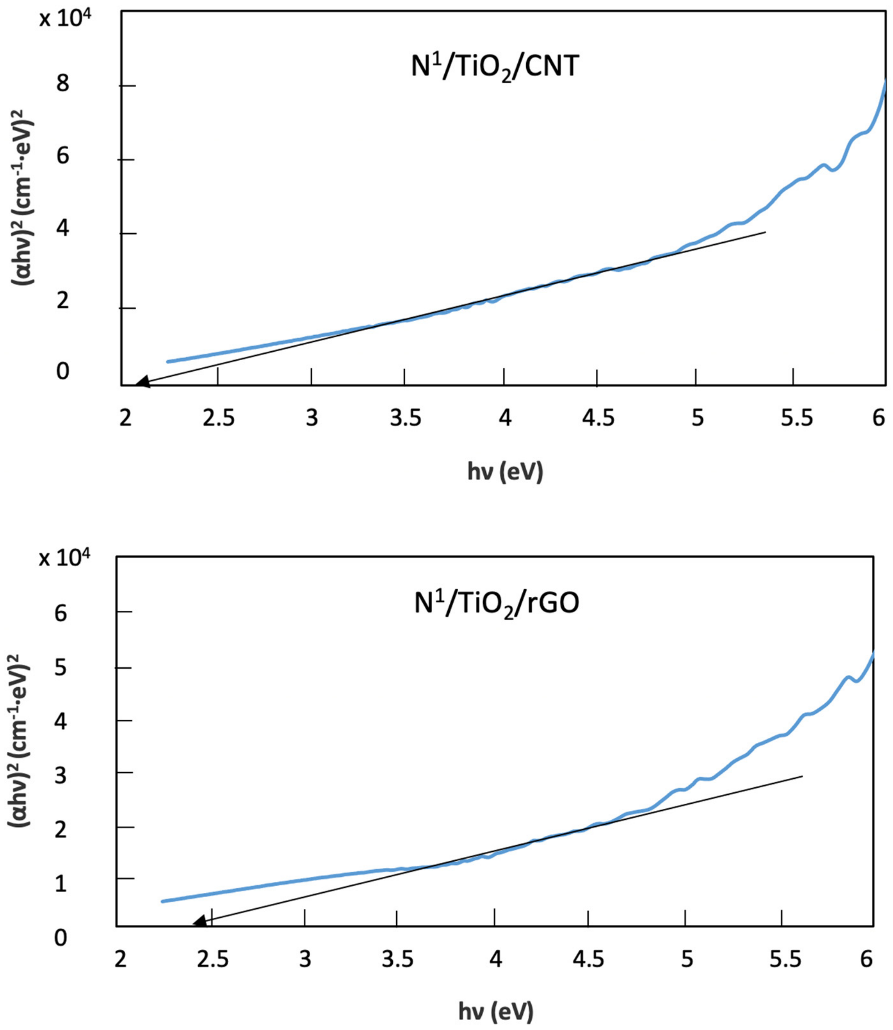

Figure S2. Tauc's plots of $N^1/TiO_2/CNT$ and $N^1/TiO_2/rGO$.
